# Supplementary material for: Undirected C-H Bond Activation in Aluminium Hydrido Enaminonates
Source: Molecules. 2023 Feb 24;28(5):2137. doi: 10.3390/molecules28052137 (PMC10004653; doi:10.3390/molecules28052137)
Supplement: Supplementary file 1 [file molecules-28-02137-s001.zip › molecules-2239976-supplementary.docx]

Undirected C-H Bond Activation in Aluminium
Hydrido Enaminonates

Chijioke Kingsley Amadi, Ufuk Atamtürk, Andreas Lichtenberg, Aida Raauf and Sanjay Mathur *

Institute of Inorganic Chemistry, Department of Chemistry, University of Cologne, Greinstr. 6, 50939 Cologne, Germany; camadi@smail.uni-koeln.de (C.K.A.); ufuk.atamtuerk01@gmail.com (U.A.); andreas.lichtenberg@uni-koeln.de (A.L.); ajamil@uni-koeln.de (A.R.).

***** Correspondence: sanjay.mathur@uni-koeln.de (S.M.)

Figure S1: ^1^H NMR Spectrum of **(ETFB)** in CDCl_3_ at RT with schematic and arbitrary numbering.....................................................................................................................................................................2

Figure S2: ^19^F NMR Spectrum of **(ETFB)** in CDCl_3_ at RT with schematic and arbitrary numbering………...3

Figure S3: ^1^H NMR Spectrum of (**H-TFB-TBA**) in CDCl_3_ at RT with schematic and arbitrary numbering…..................................................................................................................................................................4

Figure S4: ^19^F NMR Spectrum of **(H-TFB-TBA)** in CDCl_3_ at RT with schematic and arbitrary numbering......................................................................................................................................................................5

Figure S5: ^1^H NMR spectra of complex **3** recorded in C_6_D_6_. Inset shows a magnified portion of the spectrum of (**3**) to illustrate the signal due to hydride ligands attached to aluminium…………………………………...6

Figure S6: ^19^F NMR spectra of complex **3** recorded in C_6_D_6_....................................................................................7

Figure S7**.** ^19^F NMR spectra of **4b** and **4b´** recorded in C_6_D_6_ at room temperature…………………………….8

Figure S8**.** ^13^C NMR spectra of **4b** and **4b´** recorded in C_6_D_6_ at room temperature……………………………..8

Figure S9**.** ^1^H−^13^C heteronuclear multiple bond correlation (HMBC) spectra of 4b and 4b´ at room temperature………………………………………………………………..…………………………………………..9

Figure S10**.** ^1^H−^13^C Heteronuclear multiple quantum coherence (HMQC) spectra of 4b and 4b´ at room temperature……………………………………………………………………….…………………………………...9

Figure S11: Solution ^27^Al NMR spectra of complex **3** recorded in C_6_D_6_ at room temperature………………...10

Figure S12: Solution ^27^Al NMR spectra of complexes **4b and 4b´** recorded in C_6_D_6_ at room temperature………………………………………………………………………………………….....…………….10

Figure S13. FT-IR spectra of the enaminone ligand (black), compound **3** (red) and compound (blue)…………………………………………………………………………………………………………………11

Figure S14: Obtained XRD pattern of the residue **3** and **4a** after TG…………………….……….……………11

Figure S15. Representative SEM images of post/annealed thin films deposited using **4** as precursor in a CVD process.…………………………………………………………………………………………………………...….12


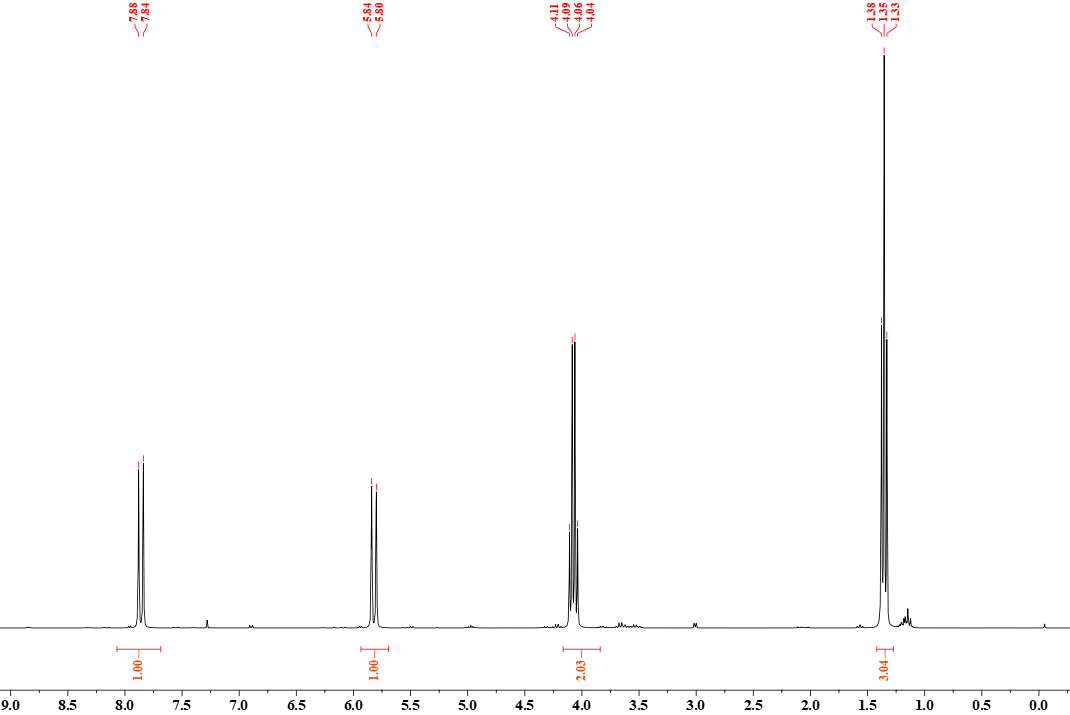


**1**

**2**

**3**

**4**

**5**

**6**

**6-H**

**5-H**

**4-H**

**3-H**

**ppm**

**Figure S1:** ^1^H NMR Spectrum of **ETFB** **1** in CDCl_3_ at RT with schematic and arbitrary numbering**.**


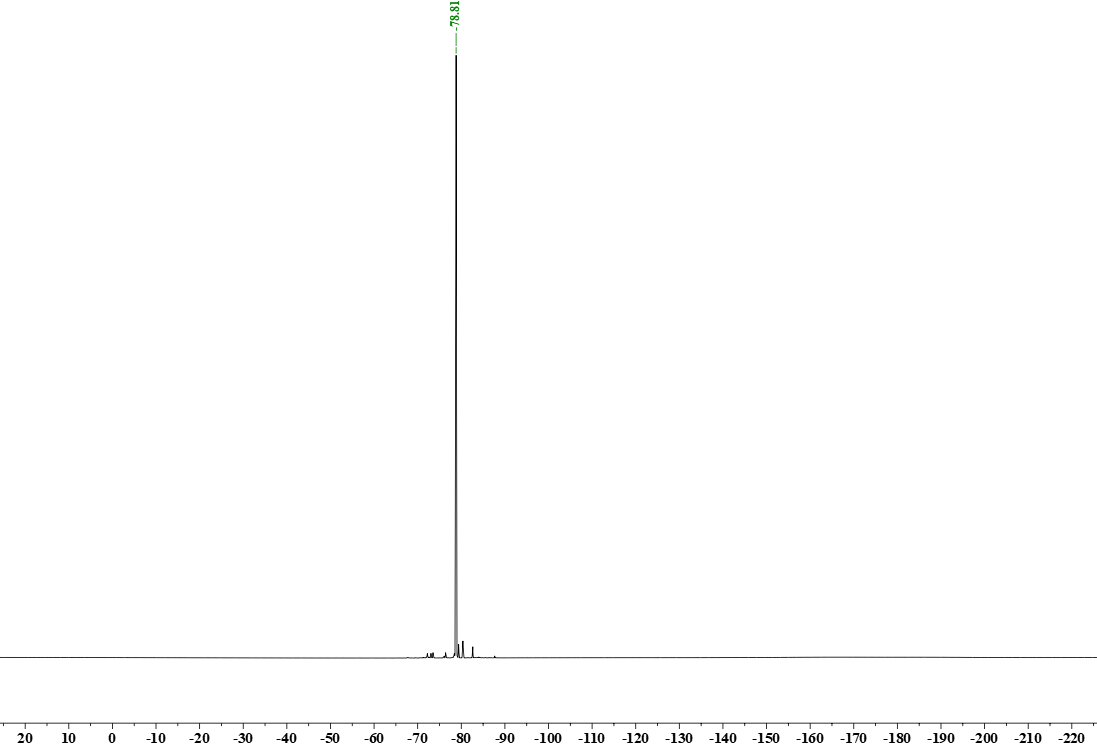


**1**

**2**

**3**

**4**

**5**

**6**

**ppm**

**1-F**

**1-F**

**Figure S2**: ^19^F NMR Spectrum of **ETFB** **1** in CDCl_3_ at RT with schematic and arbitrary numbering**.**


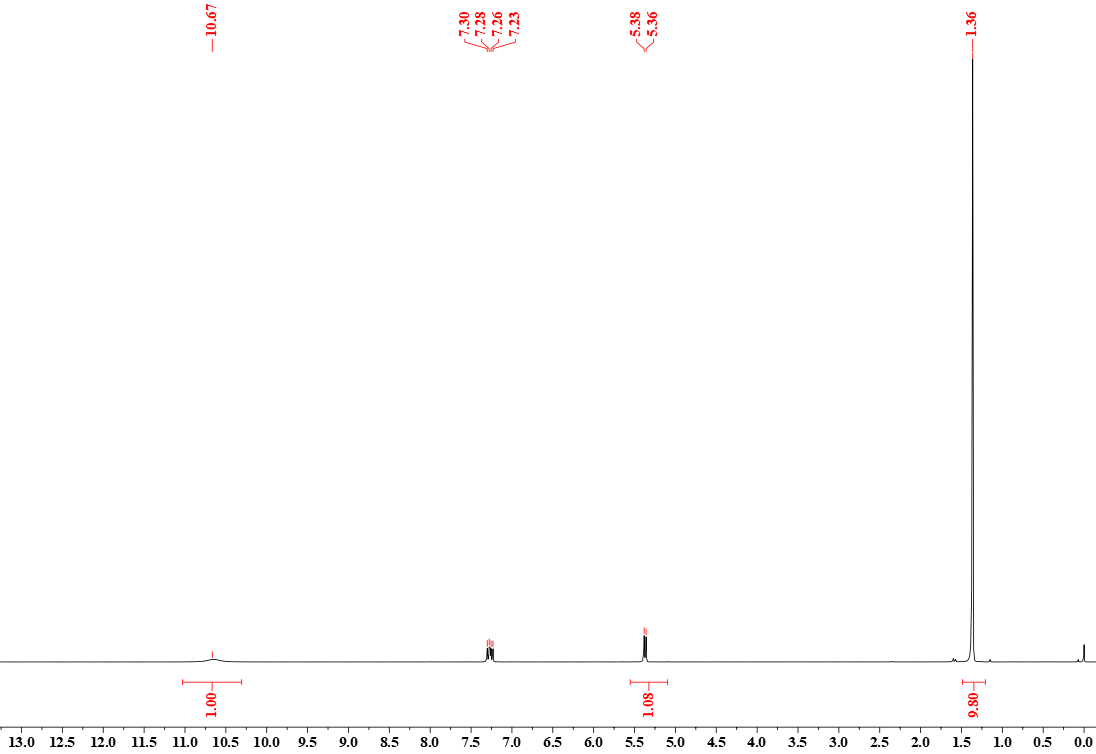


**ppm**

**1**

**2**

**3**

**4**

**5**

**NH**

**5-H**

**4-H**

**3-H**

**Figure S3**: ^1^H NMR Spectrum of **H-TFB-TBA** **2** in CDCl_3_ at RT with schematic and arbitrary numbering**.**


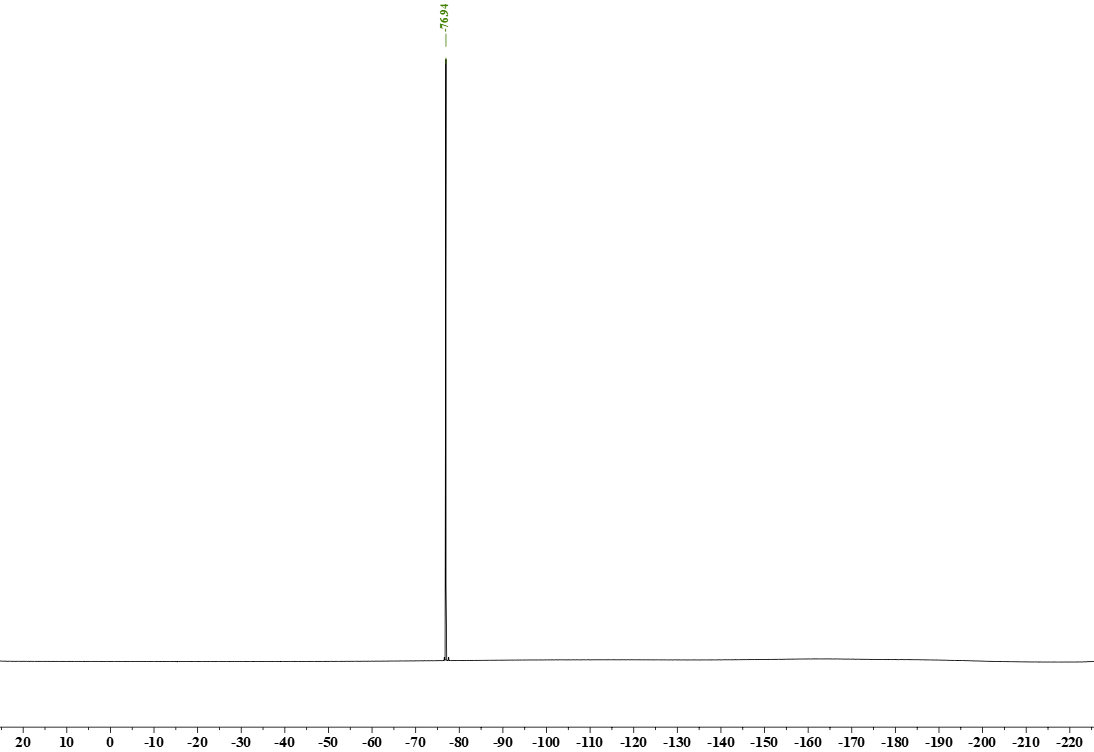


**ppm**

**1**

**2**

**3**

**4**

**5**

**1-F**

**Figure S4:** ^19^F NMR Spectrum of **H-TFB-TBA** **2** in CDCl_3_ at RT with schematic and arbitrary numbering**.**


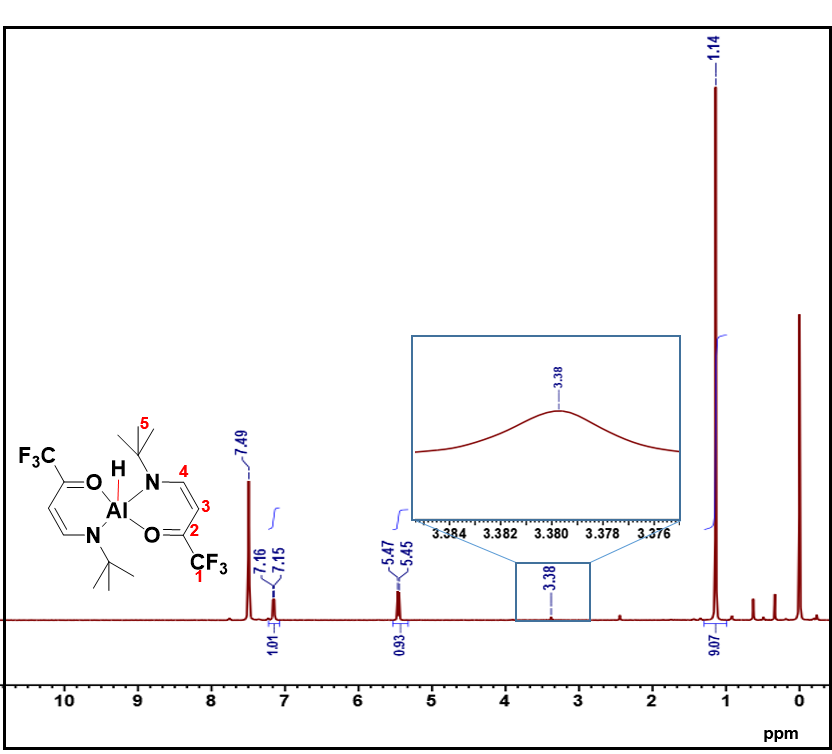


**Figure S5.** ^1^H NMR spectra of complex (**3**) recorded in C_6_D_6_. Inset shows a magnified portion of the spectrum of (**3**) to illustrate the signal due to hydride ligands attached to aluminium.


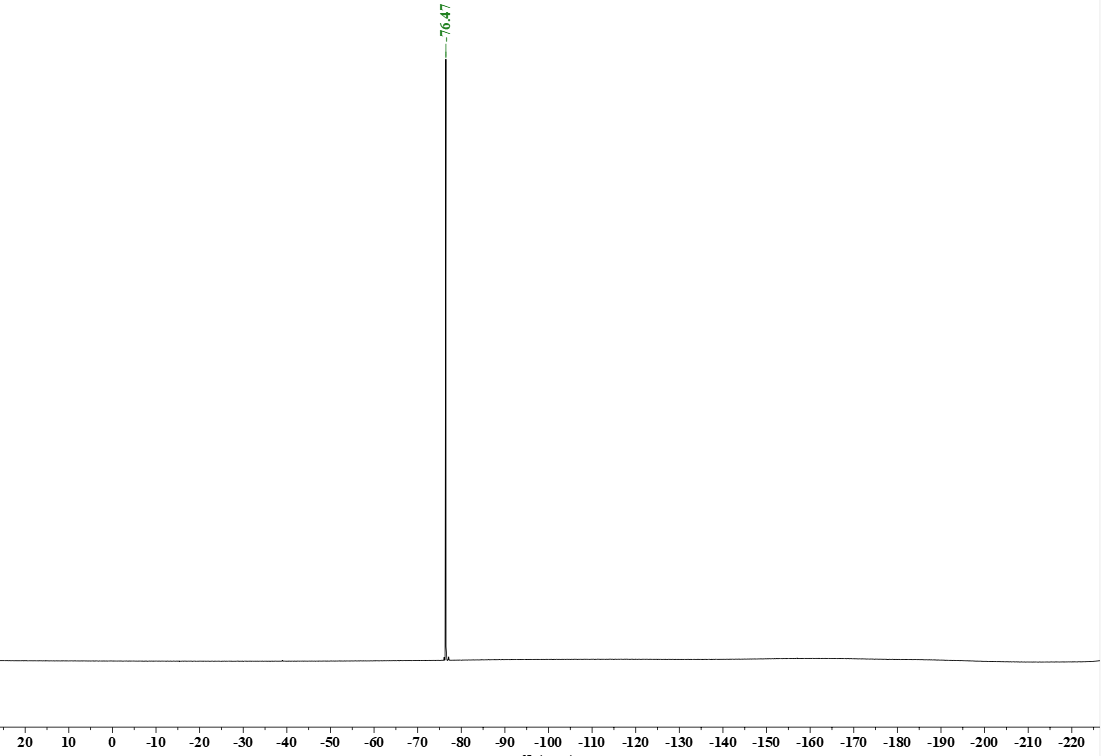


**ppm**

**3**

**5**

**4**

**1**

**1-F**

**2**

**Figure S6.** ^19^F NMR spectra of complex (**3**) recorded in C_6_D_6_.

**
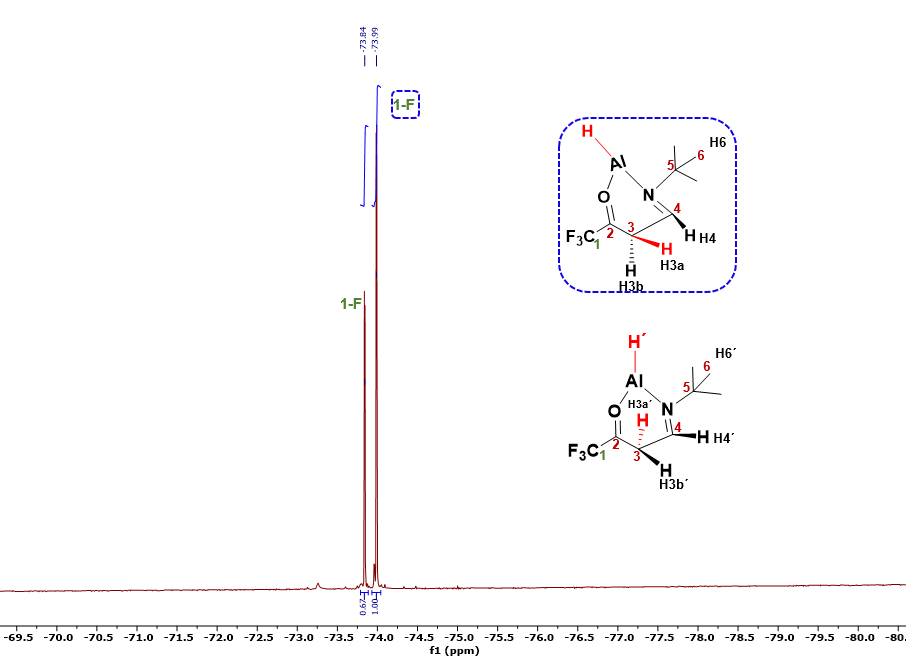
**

**Figure S7.** ^19^F NMR spectra of **4b** and **4b´** recorded in C_6_D_6_ at room temperature.

**
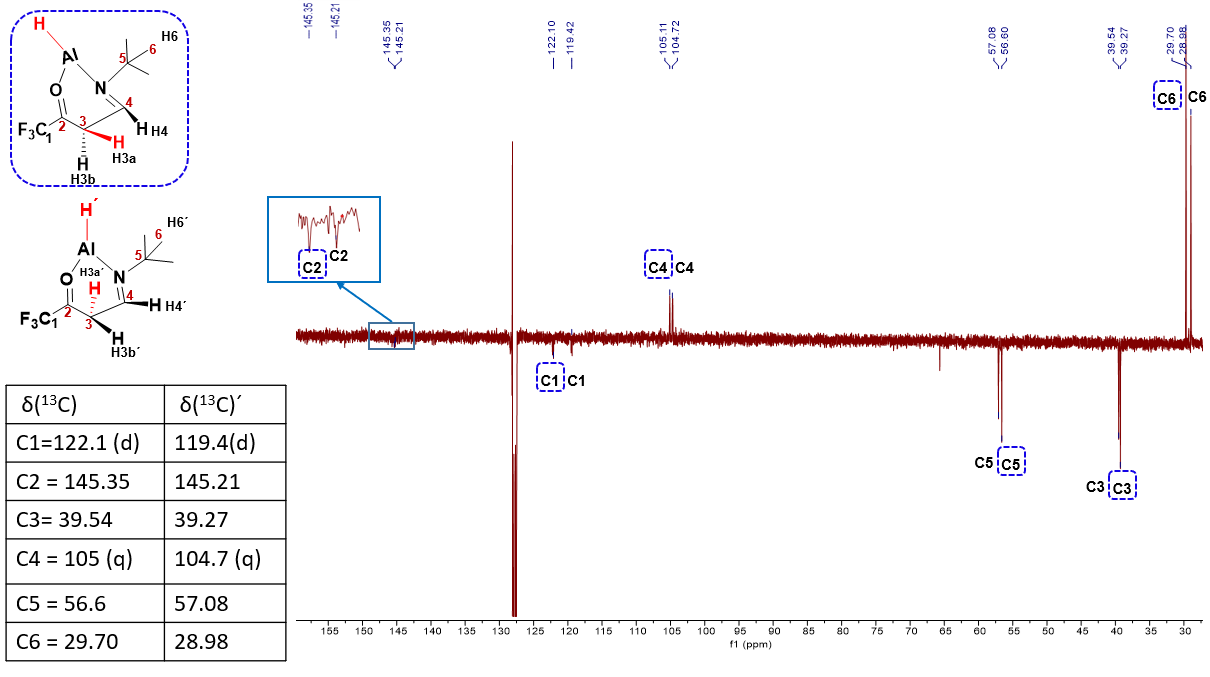
**

**Figure S8.** ^13^C NMR spectra of **4b** and **4b´** recorded in C_6_D_6_ at room temperature.

**
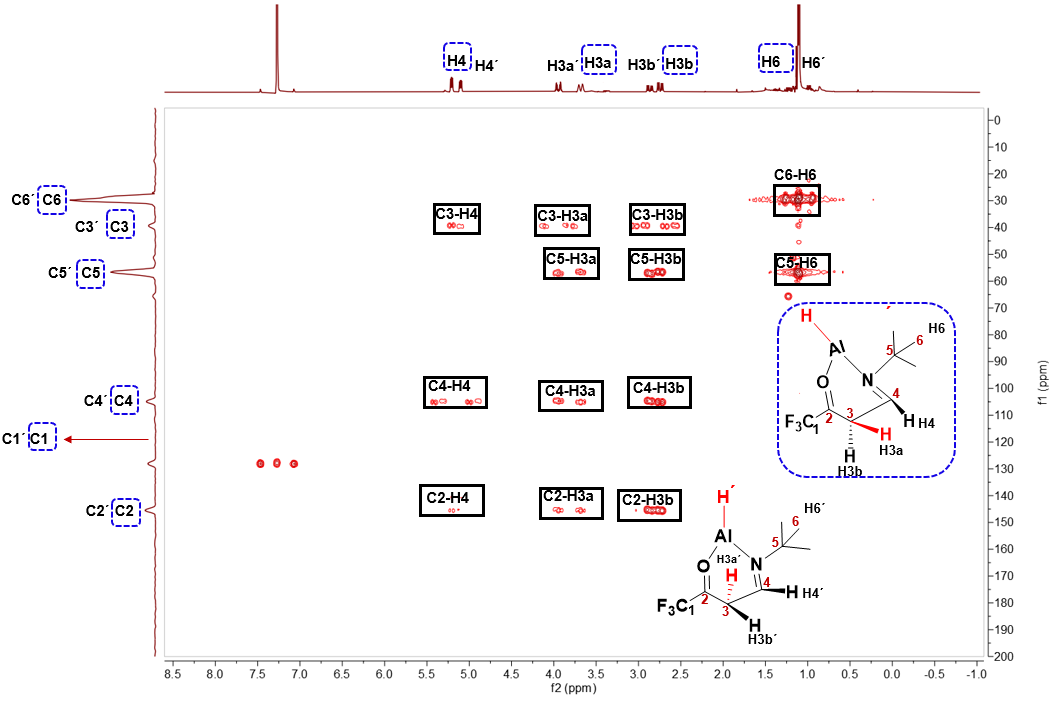
**

**Figure S9.** ^1^H−^13^C heteronuclear multiple bond correlation (HMBC) spectra of **4b** and **4b´** at room temperature.

**
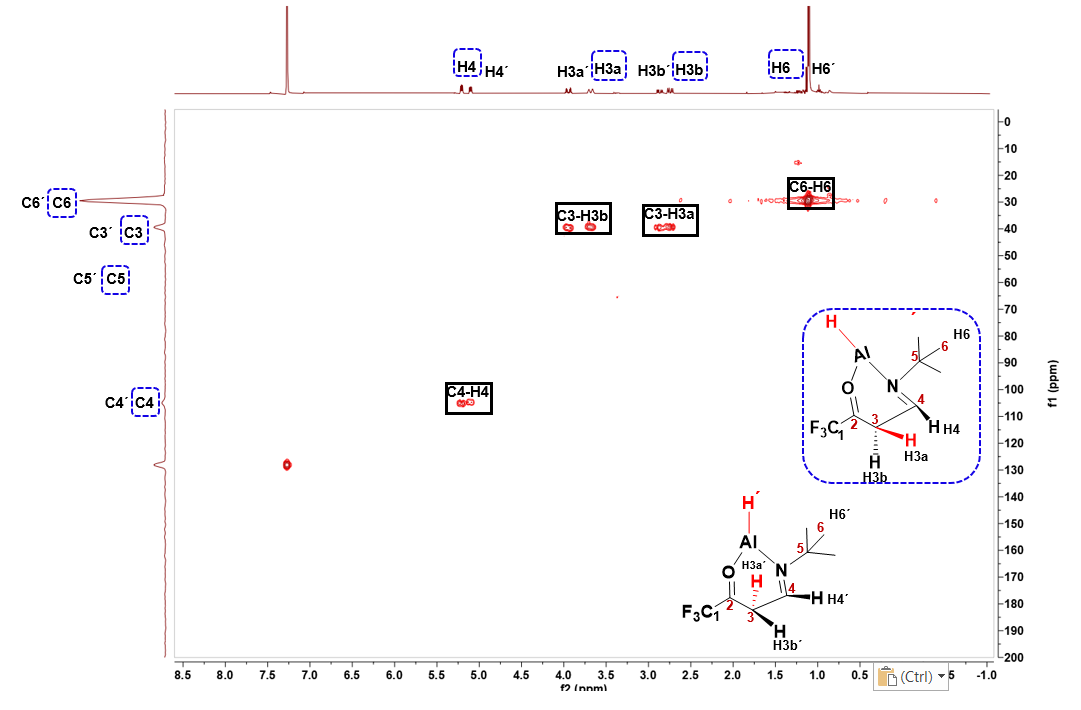
**

**Figure S10.** ^1^H−^13^C Heteronuclear multiple quantum coherence (HMQC) spectra of **4b** and **4b´** at room temperature.

**
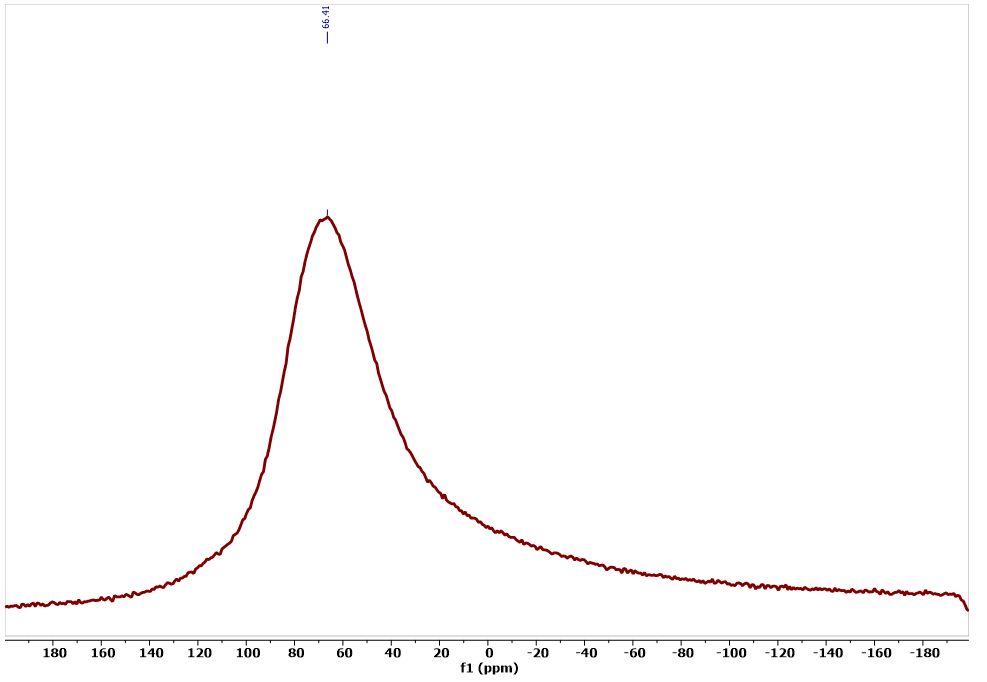
**

**Figure S11**: Solution ^27^Al NMR spectra of complex **3** recorded in C_6_D_6_ at room temperature

**
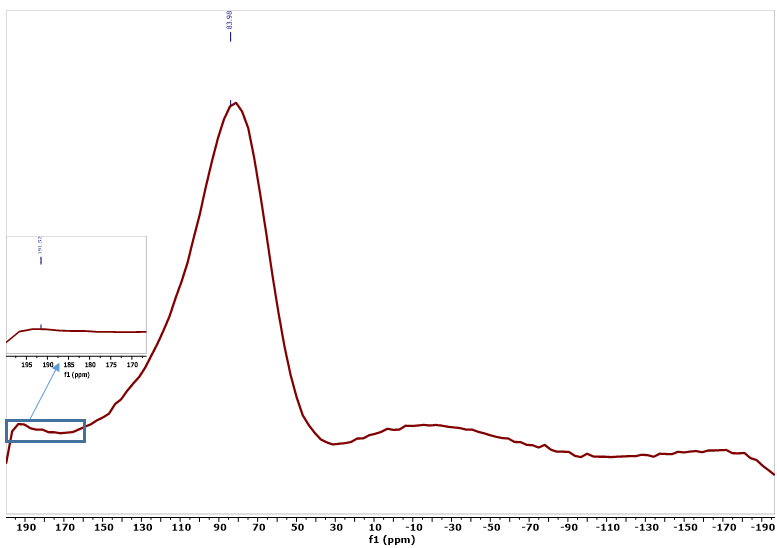
**

**Figure S12**: Solution ^27^Al NMR spectra of complexes **4b** and **4b´** recorded in C_6_D_6_ at room temperature

**Al-H**

**4b 4b´**

**Ligand**

**3**

**Wavenumber (cm^-1^)**

**Figure S13**. FT−IR spectra of the enaminone ligand (black), compound **3** (red) and compound **4b and 4b´** (blue).


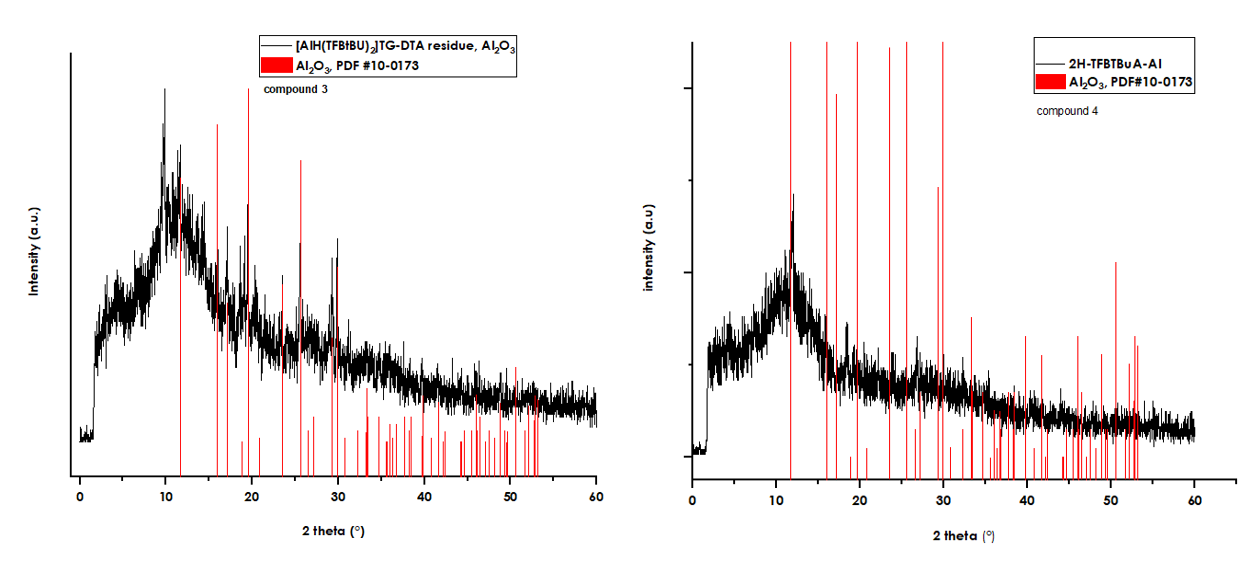


**Figure S14**: Obtained XRD pattern of the residue of **3** and **4a** after TG.


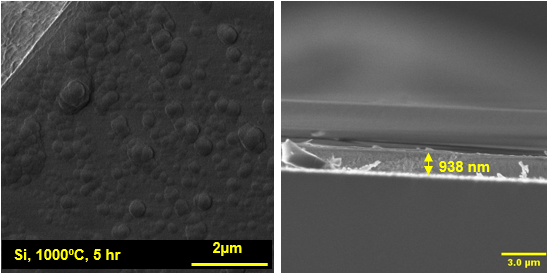


**Figure S15**. Representative SEM images of post-annealed thin films deposited using **4a** as precursor in a CVD process.
